# Spatiotemporal properties of microsaccades: Model predictions and experimental tests

**DOI:** 10.1038/srep35255

**Published:** 2016-10-14

**Authors:** Jian-Fang Zhou, Wu-Jie Yuan, Zhao Zhou

**Affiliations:** 1 College of Physics and Electronic Information, Huaibei Normal University, Huaibei 235000, China; 2 College of Information, Huaibei Normal University, Huaibei 235000, China

## Abstract

Microsaccades are involuntary and very small eye movements during fixation. Recently, the microsaccade-related neural dynamics have been extensively investigated both in experiments and by constructing neural network models. Experimentally, microsaccades also exhibit many behavioral properties. It’s well known that the behavior properties imply the underlying neural dynamical mechanisms, and so are determined by neural dynamics. The behavioral properties resulted from neural responses to microsaccades, however, are not yet understood and are rarely studied theoretically. Linking neural dynamics to behavior is one of the central goals of neuroscience. In this paper, we provide behavior predictions on spatiotemporal properties of microsaccades according to microsaccade-induced neural dynamics in a cascading network model, which includes both retinal adaptation and short-term depression (STD) at thalamocortical synapses. We also successfully give experimental tests in the statistical sense. Our results provide the first behavior description of microsaccades based on neural dynamics induced by behaving activity, and so firstly link neural dynamics to behavior of microsaccades. These results indicate strongly that the cascading adaptations play an important role in the study of microsaccades. Our work may be useful for further investigations of the microsaccadic behavioral properties and of the underlying neural dynamical mechanisms responsible for the behavioral properties.

When the eyes fixate at a stationary object, they are never completely motionless, but perform involuntary, very small eye movements. In the fixational eye movements, microsaccades are jerk-like movements^1^. It has been experimentally found that the most significant neuronal responses to fixational eye movements are generated by microsaccades^2^. So, both experimental and theoretical works have mainly focused on the role of microsaccades in neural response during fixation. Recently, neural responses induced by microsaccades have been extensively found at different levels—from neuronal activities^3^,^4^,^5^,^6^ to electroencephalogram (EEG)^7^,^8^ and functional magnetic resonance imaging (fMRI)^9^,^10^—in a number of cortical areas, including V1^3^,^9^,^10^, V2^5^,^9^,^10^, V3^10^, V4^5^, and MT^6^,^10^. Experimentally, microsaccades also exhibit many behavioral spatiotemporal properties. It’s well known that, the study of behavioral properties in life is very difficult^11^,^12^,^13^,^14^. The behavior properties are determined by neural dynamics. So, linking neural dynamics to behavior is one of the central goals of neuroscience^15^. However, the behavioral properties resulted from neural responses to microsaccades, are not yet understood and are rarely studied theoretically.

On the visual pathway, retinal adaptation has been found in experiments^16^,^17^,^18^ and so affected the microsaccade-induced neural responses^4^. Meanwhile, short-term depression (STD) has been extensively found at thalamocortical synapses from the lateral geniculate nucleus (LGN) to the primary visual cortex (V1)^19^,^20^,^21^. Previously, network models of V1 neurons with STD at thalamocortical synapses have been used to account for important response properties of cortical neurons^22^,^23^. Particularly, a recent work explored that the STD could give an alternative explanation for microsaccades in counteracting visual fading during fixation^22^.

Actually, retinal adaptation is not exclusive with STD at thalamocortical synapses. We have recently constructed a cascading network model including two levels of adaptations in ref. 24. Our computational studies have explored the impact of interplay between the two adaptations on network dynamics and found various rich dynamics. Particularly, the cascading adaptations give rise to fast and sharp responses to microsaccades. The fast and sharp responses exhibit small timescales of neural dynamics. By linking the neural dynamics to behavior, the timescales of behavior about microsaccades are expected to be explored. In this paper, we firstly predict two behavioral relations about microsaccadic magnitudes and time intervals, according to the timescales of simulated neural dynamics in the cascading model. Secondly, the behavioral predictions are experimentally verified in the statistical sense.

## Results

### Model predictions

**Figure 1:**
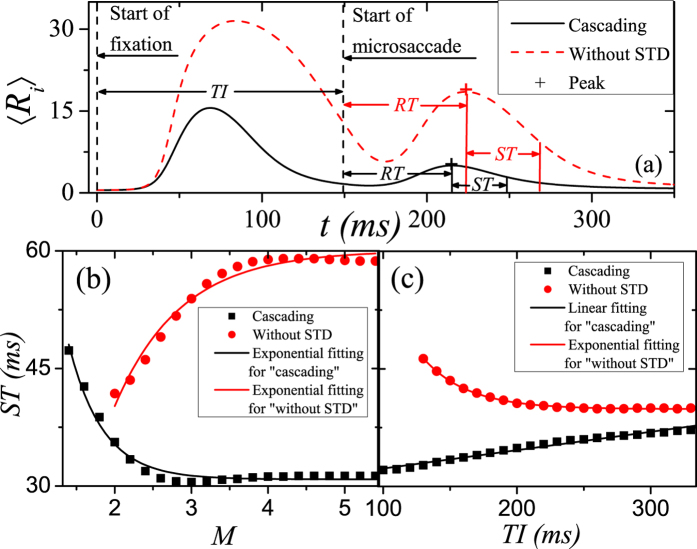
(Adapted from ref. 24) Comparison of V1 responsive timescale induced by microsaccade in cascading-adaptation network model and in the absence of STD. (**a**) The network-averaged firing rate 〈*R*_*i*_〉 evoked by the fixation and a microsaccade which occurs at the time interval *TI* after the onset of the fixation. In (**a**), solid verticals denote responsive peaks and half-high values between peak and baseline. The sustaining time *ST* of responsiveness as a function of microsaccade magnitude *M* (**b**) and fixation-microsaccade time interval *TI* (**c**). In (**b**,**c**), the lines denote exponential and linear fittings. Here, we give *TI* = 150 ms (**a**,**b**) and *M* = 2.2 (**a**,**c**).

We firstly compute V1 neural responses to microsaccades in the section. Here, we study the neural responses to an independent (isolated) microsaccade with different microsaccadic size and fixation-microsaccade interval. In Fig. 1(a), we plot the V1 network-averaged firing rate 〈*R*_*i*_〉 induced by fixation and microsaccade with fixation-microsaccade time interval *TI*. When successive microsaccades occur, the time interval *TI* can also be regarded as time interval between two neighbor microsaccades. In order to focus on the effect of STD, we compare responsive time (*RT*) and sustaining time (*ST*) of the neural activity related to microsaccades in the cascading-adaptation model and in the absence of STD at thalamocortical synapses. As shown in Fig. 1(a), the *RT* is thought as the time interval from start of microsaccade to responsive peak after microsaccade. The *ST* is regarded as the time interval from responsive peak to half-high value before decaying to baseline after the microsaccade.

Particularly, we compare the *ST* of response in the cascading-adaptation model and in the absence of STD. It is noteworthy that, Fig. 1(b,c) clearly show that changes of the *ST* as a function of microsaccadic size *M* and of fixation-microsaccade interval *TI* in cascading-adaptation model and those in the absence of STD display opposite trends. The *ST* of responsiveness monotonously decreases (increases) as the increasing of *M* (*TI*) (within the small region) in the cascading-adaptation model, while it monotonously increases (decreases) in the absence of STD. The opposite changes of *ST* have been explored to be contributed to the strong STD^24^.

**Figure 2:**
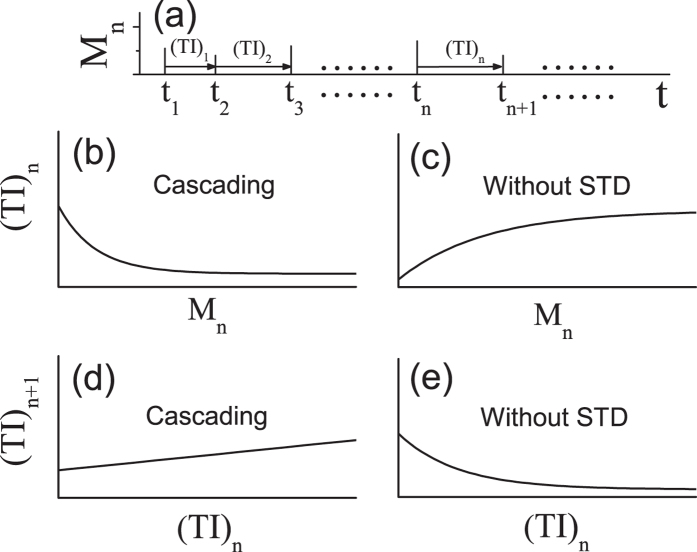
Predictions of the relations of microsaccadic magnitudes and intervals according to different network models. (**a**) Schematics of microsaccadic magnitude *M*_*n*_ and interval (*TI*)_*n*_. Here, *M*_*n*_ denotes the *n*-th microsaccadic magnitude (black tick) at the time *t* = *t*_*n*_. The *n*-th microsaccadic interval (*TI*)_*n*_ is defined by *t*_*n*+1_ − *t*_*n*_. The predicted relations between *M*_*n*_ and (*TI*)_*n*_ in cascading-adaptation network (**b**) and in the absence of STD (**c**). The predicted relations between (*TI*)_*n*_ and (*TI*)_*n*+1_ in cascading-adaptation network (**d**) and in the absence of STD (**e**). In (**b**,**c**), the lines of relations come from the exponential fittings of simulated results in Fig. 1(b). In (**d**,**e**), the lines of relations come respectively from the linear and exponential fittings of simulated results in Fig. 1(c).

Since microsaccades can be required for counteracting visual fading during fixation^4^, the *ST* of visual neural response induced by microsaccades can reflect the microsaccadic time interval *TI*. In Fig. 2(a), we give schematics of the *n*-th microsaccadic magnitude *M*_*n*_ and interval (*TI*)_*n*_. According to Fig. 1(b), we can give a behavioral prediction of the relation between microsaccadic magnitudes and intervals in Fig. 2(b). It can be seen that the microsaccadic time interval decays exponentially with the increase of microsaccadic magnitude. This indicates that the larger the microsaccadic magnitude (within the small region), the shorter the microsaccadic time interval. Namely, a smaller micacrosaccdic interval could follow the previous microsaccade with a larger size (in the sense of statistical average). For comparison, we give in Fig. 2(c) the opposite relation in the absence of STD. Similarly, according to Fig. 1(c) we can also give another behavioral prediction about relation of two adjacent microsaccadic intervals, shown in Fig. 2(d). We can see that STD contributes to a slightly linear positive relation of two adjacent microsaccadic intervals. Namely, if the microsaccadic time interval is large, the next microsacadic time interval could also tend to be large (in the sense of statistical average), and vice versa. However, in the absence of STD the behavioral relation exhibits the opposite trend in Fig. 2(e). Therefore, the two predictions in Fig. 2(b,d) can be used to experimentally verify the important role of the cascading adaptations in neural response to microsaccades by finding the evidence for the above relationships.

### Experimental tests

**Figure 3:**
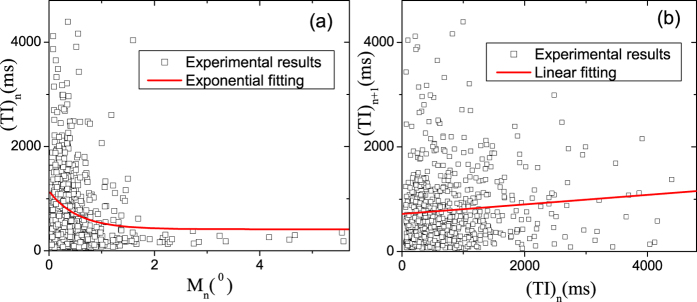
Experimental data for testing predictions. Experimental results about the relation between *M*_*n*_ and (*TI*)_*n*_ (**a**), and about the relation between (*TI*)_*n*_ and (*TI*)_*n*+1_ (**b**). In (**a**,**b**), the lines are given by the exponential and linear fittings of experimental data, respectively.

In order to test our predictions, we show behavioral properties about microsaccadic magnitudes and time intervals, which are recorded in experiments. In Fig. 3(a,b), the experimentally behavioral relations about microsaccades consist with (in the statistical sense) the above theoretical predictions in the cascading-adaptation model, shown in Fig. 2(b,d). Meanwhile, we find that they are qualitatively opposite to the results in the absence of STD (see Fig. 2(c,e)). For involuntary microsaccades, the exhibiting behavioral relations of their magnitudes and time intervals could be required for the neural spontaneous responses induced by STD. Maybe, this result can be used to explain why microsaccades (including magnitudes and time intervals) happen involuntarily, not depend on human desires. This experimental verification indicates strongly that the cascading adaptations including STD, play a very important role in neural responses to microsaccades, and so in behavioral properties of microsaccades.

## Discussion

By using a cascading-adaptation model including STD, we theoretically studied the sustaining time of neural responses to microsaccades for different microsaccadic sizes and microsaccadic time intervals. According to the timescales of sustaining responses, we predicted two behavioral relations about spatiotemporal properties of microsaccades. Experimentally, we verified the behavior predictions in the statistical sense. Our results suggest strongly that the cascading adaptations including both retinal adaptation and STD, play an important role in neural responses to microsaccades, which is responsible for behavioral properties of microsaccades.

For linking neural dynamics to behavior, a major impediment is the large difference between neurophysiological and behavioral timescales^15^. In our work, the timescales are different between neural dynamics and behavior. The neural dynamical timescales were weakly, but significantly, correlated with those of microsaccadic behavior. In the future work, we will try to find a mechanism to reduce the gap in timescales. The work presented in this paper serves as a foundation for further investigation in the future.

To concentrate on the effects of STD on microsaccades, we here kept our model very simple. The current model omitted some properties that thalamo-cortical network is known to exhibit^25^. Firstly, we entirely adopted the excitatory input from thalamocortical relay cells, and did not consider thalamic reticular (inhibitory) neurons in LGN^25^. Secondly, no account has been taken for recurrent intracortical connections between excitatory and inhibitory neurons^26^,^27^. Thirdly, our model does not include corticothalamic feedback^28^. Finally, another important type of synaptic plasticity, spike-timing-dependent plasticity (STDP)^29^,^30^, which has been found in visual cortex *in vivo*^31^,^32^,^33^, was not considered.

In the future, interplay between the different forms of synaptic plasticity (e.g., STD and STDP) seen in thalamocortical and intracortical neurons, combining the rich dynamics of recurrent intrathalamic and intracortical excitation and inhibition, as well as corticothalamic feedback, will make the study of microsaccades in more realistically connected models both interesting and challenging. The further investigations are expected to understand more behavioral properties of microsaccades (for review, see ref. 2).

Generally, our work is the first to give behavior description of microsaccades based on neural dynamics, and so firstly links neural dynamics to behavior of microsaccades. The modeling study may provide a starting point for exploring theoretically the microsaccadic behavior by simulating neural responses to microsaccades. Our study can provide a useful tip for the understanding of more behavioral properties of microsaccades in the future.

## Methods

**Figure 4:**
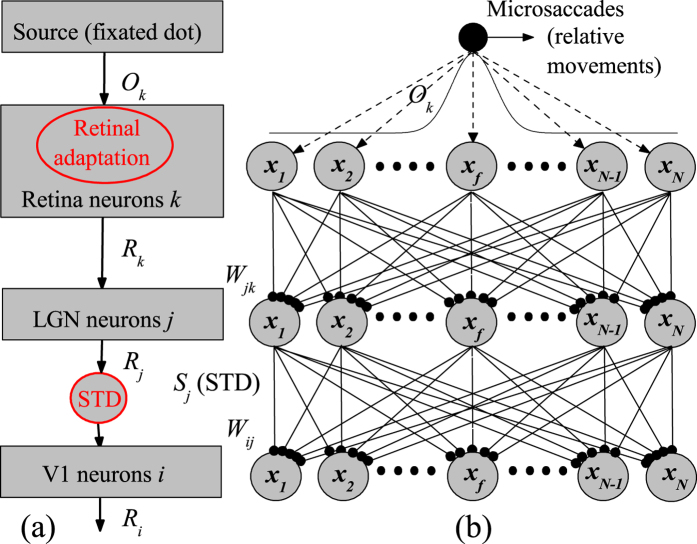
(Adapted from ref. 24) The feedforward cascading-adaptation network model including retinal adaptation and STD during fixation with microsaccades. (**a**) The schematic sketch of visual pathway. The received optical strengths *O*_*k*_ from the fixated dot induce the firings in retina cells, the firings with rate *R*_*k*_ in retina are directly projected to LGN, then the produced firings with rate *R*_*j*_ in LGN are straightly projected to V1, and finally, the firings with rate *R*_*i*_ are generated in V1. (**b**) Illustration of the feedforward network structure. There is the same number of *N* neurons in retina, LGN and V1. These neurons in retina, LGN and V1 are all labeled and arranged corresponding to the center positions *x*_*k*_, *x*_*j*_ and *x*_*i*_ of their receptive fields distributed uniformly in the ranges from −*L* to *L*, respectively. *x*_*f*_ denotes the position of the fixated point. The retina cells are connected to LGN by neural synapses with linking weights *W*_*jk*_. The LGN are then connected to V1 by thalamocortical synapses with linking weights *W*_*ij*_ and with synaptic strengths *S*_*j*_, which are subjected to the modification by STD. The microsaccades during fixation can be regarded as instantaneous relative movements of the fixated dot. In order to eliminate the effect of boundaries due to limited network scale, periodic boundary conditions are applied to the three layers of the network (thus to the couplings between layers and the input curve *O*_*k*_).

According to visual passway^34^,^35^, we proposed a feedforward network model with cascading adaptations in ref. 24, including both retinal adaptation and STD at thalamocortical synapses. The model is composed of firing rate neurons. As shown in Fig. 4(a), the received optical strength *O*_*k*_ gives rise to the neuron fires with rate *R*_*k*_ by retinal adaptation in retinal cell *k*. The firings *R*_*k*_ are directly projected to LGN neuron *j*. Then, the firings with rate *R*_*j*_ in neuron *j* are straightly projected to V1 neuron *i* by synapses with STD.

More details of the cascading network structure are shown in Fig. 4(b) and described in its caption. The neural network model is composed of excitatory neurons, described by membrane potential and firing rate. In LGN neuron *j* and V1 neuron *i*, the dynamics of membrane potential *V*_*j*_, *V*_*i*_ and their firing rates *R*_*j*_, *R*_*i*_ can be described by


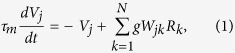



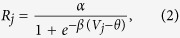



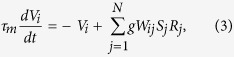



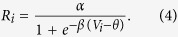


Additionally, the firing rate *R*_*k*_ in retinal cell *k* can be described by


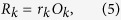


where *r*_*k*_ is an adaptation factor of responsive transition from received optical strength *O*_*k*_ (coming from fixated dot) to firing rate *R*_*k*_. In Eq. 3, the synaptic strength *S*_*j*_ is subjected to the following STD mechanism^36^,^37^:


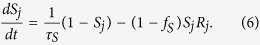


Similar to the property of STD, we can describe *r*_*k*_ in Eq. 5 by using the adaptation scheme,


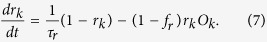


Because of receptive fields (Gaussian filters)^38^,^39^,^40^ in retinal, LGN and V1 neurons, the received optical strength *O*_*k*_ is here assumed to be the Gaussian profile: 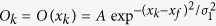. Meanwhile, the spatial connecting weights *W*_*jk*_ and *W*_*ij*_ follow the Gaussian tuning curves^22^,^35^: 
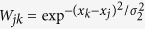
 and 
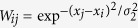
.

Since microsaccades happen rather quickly, they are modeled by instantaneous relative displacement *M* of the curve *O*_*k*_ on the one dimensional straight direction of retinal neural positions. In our simulations, the parameters are given by *g* = 1.8, *τ*_*m*_ = 30 ms, *α* = 200, *β* = 1, *θ* = 6, *τ*_*S*_ = *τ*_*r*_ = 200 ms, *f*_*S*_ = *f*_*r*_ = 0.75, *A* = 60, *σ*_1_ = *σ*_2_ = 1.5, *N* = 1000 and *L* = 10. The main results, however, do not sensitively depend on these parameters. Choosing different parameter values does not alter the qualitative results.

In experiments, microsaccades are recorded when a small red point in the middle of screen is fixated with the background of homogeneous gray. The viewing distance to screen was 60 cm. Data of 5 subjects are included. Each fixation trial lasts 10020 ms.

## Additional Information

**How to cite this article**: Zhou, J.-F. *et al*. Spatiotemporal properties of microsaccades: Model predictions and experimental tests. *Sci. Rep.* **6**, 35255; doi: 10.1038/srep35255 (2016).
